# Autoimmunity in Pulmonary Arterial Hypertension: Evidence for Local Immunoglobulin Production

**DOI:** 10.3389/fcvm.2021.680109

**Published:** 2021-09-21

**Authors:** Ting Shu, Yanjiang Xing, Jing Wang

**Affiliations:** ^1^State Key Laboratory of Medical Molecular Biology, Department of Pathophysiology, Institute of Basic Medical Sciences, Chinese Academy of Medical Sciences, Peking Union Medical College, Beijing, China; ^2^State Key Laboratory of Medical Molecular Biology, Department of Physiology, Institute of Basic Medical Sciences, Chinese Academy of Medical Sciences, Peking Union Medical College, Beijing, China

**Keywords:** pulmonary arterial hypertension, adaptive response, auto-antibody, auto-antigen, immunoglobulins

## Abstract

Pulmonary arterial hypertension (PAH) is a progressive life-threatening disease. The notion that autoimmunity is associated with PAH is widely recognized by the observations that patients with connective tissue diseases or virus infections are more susceptible to PAH. However, growing evidence supports that the patients with idiopathic PAH (IPAH) with no autoimmune diseases also have auto-antibodies. Anti-inflammatory therapy shows less help in decreasing auto-antibodies, therefore, elucidating the process of immunoglobulin production is in great need. Maladaptive immune response in lung tissues is considered implicating in the local auto-antibodies production in patients with IPAH. In this review, we will discuss the specific cell types involved in the lung *in situ* immune response, the potential auto-antigens, and the contribution of local immunoglobulin production in PAH development, providing a theoretical basis for drug development and precise treatment in patients with PAH.

## Introduction

Pulmonary arterial hypertension (PAH) is characterized by pulmonary vascular remodeling in pathology, leading to the elevation of mean pulmonary arterial pressure. Pulmonary vascular remodeling is seen as a result of perivascular inflammatory cells infiltration and pulmonary arterial wall dysfunctions (1, 2). The inflammatory cells infiltration is considered as both the cause and the consequence of pulmonary vascular remodeling. Innate response and adaptive response are found in the lung tissues of clinic PAH and experimental PH (1–4). The innate response is participated by macrophages/monocytes (5), mast cells (6), neutrophils (7), etc. These cells are recruited from peripheral blood and infiltrated around pulmonary vessels. Macrophages, especially interstitial macrophages, function by releasing cytokines or chemokines, such as IL-6, TNFα, and CCL2. TNFα suppressed BMPRII expression in pulmonary arterial endothelial cells (PAECs) and pulmonary arterial smooth muscle cells (PASMCs) (8). IL-6 promotes PASMC proliferation and activates fibroblasts (9). CCL2 will recruit more inflammatory cells into the lung tissues and promotes crosstalk between macrophages and PASMCs (10). Mast cells and neutrophils belonged to the granulocytes, which will degranulate once activated. Granule content, such as myeloperoxidase (MPO) in neutrophils or protease in mast cells, contributes to pulmonary vascular remodeling (6, 11). Antigen-presenting cells (APCs) will also be attracted by chemokines in the early stage (12). The identification of dendritic cells (DCs) revealed the link between the innate response and adaptive response (13). Adaptive immunity is participated by T cells and B cells. APCs mediate T cell differentiating into subtypes, such as helper T cells or cytotoxic T lymphocytes. Furthermore, T cells interact with B cells and promote B-cell maturation. Moreover, auto-antibodies with atopy are produced, even in the patients with idiopathic PAH (IPAH) without a diagnosis of autoimmune diseases (14). This research suggests the local adaptive response in IPAH lung. The innate response has been recognized for decades (15); however, the role of adaptive response is reported in recent years. This review aimed to update the contribution of adaptive immune cells in patients with experimental PH and IPAH, summarize the potential target auto-antigens, discuss the types and functions of locally produced immunoglobulins, and provide promising therapeutic targets for clinic treatment.

## Immune Cells Involved in Adaptive Immunity

In 2005, Dr. Nicolls had hypothesized the *in situ* adaptive immune response and immunoglobulin generation in the IPAH lung tissues without direct evidence (16). Decades later, the key components involved in this hypothesis have been reported. The concept of local adaptive response is that pulmonary vascular injury leads to auto-antigens exposure, which are phagocytized by DCs and then presented to T cells; T cells are activated and interact with B cells, leading to B-cell antibody class-switching recombination and immunoglobulins production (1, 16). In this process, DCs, T cells, and B cells play essential roles.

### Dendritic Cells

The infiltration of DCs is observed in both IPAH and experimental pulmonary hypertension (PH) (17). Infiltrated DCs showed different gene expression signatures among the different species, which have been discussed in a previous review (18). In IPAH, perivascular DCs exhibit CD1a^−^, for rat PH, the signature is OX-62^+^ (17). This signature indicated the perivascular DCs is immature, possessing the ability for antigens presentation (17). Immature DCs are also considered as steady state and can be classified as conventional DCs (cDCs) and plasmacytoid DCs (pDCs) (13, 18). cDCs raise more attention as they show a higher frequency compared with pDCs (18). cDCs have two subsets, among which the conventional DCs subtype 2 (cDC2s) is the major population in both blood and lung tissues. The cDC2s are highly expressed MHCII, while cDC1s are superior in MHCI expression (19). The MHCI/II expressed in cDCs suggests its power in presenting the antigens. The previous studies show that in the patients with IPAH, cDCs are decreased in the blood (20) but increased in the lung tissues (21). This observation can be explained by the infiltration and retention of DCs in lung tissue. CCR7 is crucial in DCs recruitment to lymph-vessel (22, 23). CCR7 deficiency resulted in the failure of DCs homing, eventually being accumulated in the lung tissue (12). The role of cDCs is predicted by Tnfaip3/A20 deficient mice in which cDCs are activated through NF-κB signaling (24). Activated cDCs increased perivascular inflammation and subsequently aggravated PH in mice (24), suggesting its detrimental role in PAH/PH development. The infiltration of pDCs in IPAH lung tissues was first reported in 2018 (21). As a lower frequency subset, pDCs were captured by single-cell sequencing and identification in lung tissues of the patients with IPAH. Increased pDCs number was confirmed by flow cytometry and its location was revealed by staining. Although we have observed pDCs accumulation around pulmonary vessels in IPAH (21), it is hard to clarify its function as a lack of experimental evidence. Based on the function of pDCs in other tissues (25), we predicted pDCs is crucial in antivirus as it is capable in expressing interferon gene signature (such as IFN-γ, CXCL4, and CXCL10) (21, 25). These cytokines and chemokines suggest their unique pattern in cross-linking with T cells and leukocytes activation (26).

### T Cells

Naïve T cells that received antigen presentation by DCs will be activated and differentiate into CD4^+^ T cells or CD8^+^ T cells. Different DCs subtypes have the variant capacity in stimulating T-cells differentiation. cDC2s are more powerful in promoting CD4^+^ T cells, while cDC1s are superior in activating CD8^+^ T cells (19, 27). The microenvironments surrounded the cross-presentation site between DCs and naïve T cells decide the differentiation destination of T cells. Conventionally, CD4^+^T cells are divided into T helper 1 (Th1), Th2, Th17, and regulatory T cells (Treg) (28). Th1 polarization is triggered by IFN-γ, IL-12, and IL-18, and suppressed by IL-4 and TGF-β (29). Th1 cells release Th1 cytokines, such as IL-12, IFN-γ, and TNF-α, however, their contributions in clinic PAH and experimental PH are not reported clearly. Th2 polarization is induced by IL-4 and suppressed by IFN-γ (29, 30). Th2 cytokines include IL-4, IL-5, and IL-13 (30). Th2 cells infiltration and Th2 cytokines production are observed in the lung tissues of patients with IPAH and experimental subjects (31, 32). The previous studies have demonstrated that Th2 promotes PASMCs proliferation through IL-4 and IL-13 releasing (31, 32). Moreover, Th2 cells and Th2 cytokines (IL-4 and IL-5) are capable of the activation of B cells, and it facilitates immunoglobulin class-switching in B cells (33–35). Th17 polarization is stimulated by IL-1β, IL-6, IL-23, and TGF-β. Th17 cytokines are featured as IL-17 and GM-CSF. Th17 cells infiltration and IL-17 elevation are observed in the clinic and experimental PAH/PH (36, 37). Over-expressing IL-17 or blocking IL-17 in mice regulates the PH development through directly affected PAECs and PASMCs function (37). T cells that received IL-2, IL-10, and TGF-β stimulation will be polarized into Treg (28). Treg is well-known as a suppressor for the inflammatory cells in many diseases (38). In PAH/PH development, Treg suppresses vascular inflammation and alleviates PH development. These functions are observed as rats that lack Treg are more susceptible to PH (39–41), and reconstituting Treg to hypoxic mice protected against PH development (42). In patients with IPAH, circulating Treg portion increased and exhibited aberrant subtypes compared with control subjects (43–45), whether these changes are seen in lung tissues need further exploration. CD8^+^T cells are activated by cDC1s (19, 27). In patients with IPAH, circulating CD8^+^ T cells portion decreased compared with control subjects (46), but increased in PAH lung tissues, especially in obstructed sites (46, 47). Single-cell sequencing identified increased CD8^+^ T cells proportion in lung tissues of patients with IPAH (21), however, its role in PAH development needs more experimental exploration. T cells that expressed T-cell receptor (TCR) and received DCs cross-presentation (as described above) belong to the αβT cells. The other subset of T cells without TCR are named as γδT-cells (48). The presence of γδT-cells is first identified in 2018 (21). Dr. Marsh showed increased γδT-cells proportion in the lung tissues of patients with IPAH (21). The γδT-cells response is independent of antigen presentation by DCs, therefore γδT-cells belong to the innate immune (48).

### B Cells

The B cells are the core components in adaptive response and immunoglobulins production. The infiltration of B cells in lung tissues is seen in the patients with IPAH and experimental PH subjects (49, 50). Deletion of B cells protected rats against MCT-induced PH development (50). In adaptive response, B cells are activated by cross-linking with CD4^+^T cells. The activated B cells are named plasma cells (51), as they possess the ability to produce immunoglobulins. Plasma cells are detected in blood and lung tissues of patients with IPAH (51, 52), supporting the notion of auto-antibody production in the patients with IPAH. Immunoglobulin production in B cells is a complicated process, as B cells will experience the VDJ recombination and the antibody class-switching recombination. Conventionally, naïve B cells are producing IgM. Once being activated, B cells switch the heavy chain constant region from IgM to IgG, IgA, or IgE, without changing the antigen-binding site (53). Cytokines released by Th play crucial roles in mediating class-switching. IL-4 released by Th2 prompts the B cells to switch into IgG and IgE isotype (54). IFN-γ released by Th1 promotes IgG class-switching. TGF-β and IL-5 participate in IgA isotype class-switching (26, 34). Although T-cell independent pathways are also involved in B-cell class-switching, the present focus of T cells could be expanded into T cell-mediated B-cell interactions. Dr. Blum analyzed the circulating B cells in the blood of the patients with IPAH through single-cell sequencing and found that these cells are increased in number and changed in transcript signature (51). The observation of mature antibody production suggested the class-switching occurred in plasmablasts in patients with IPAH. In this process, different immunoglobulin isotypes might be produced (51). Another study found B-cell activation in circulating blood, indicated by increased Bruton's tyrosine kinase (BTK) expression in B cells from the patients with IPAH, suggesting the enhanced BCR signaling in these patients (55).

### Tertiary Lymphoid Organs

**Figure 1:**
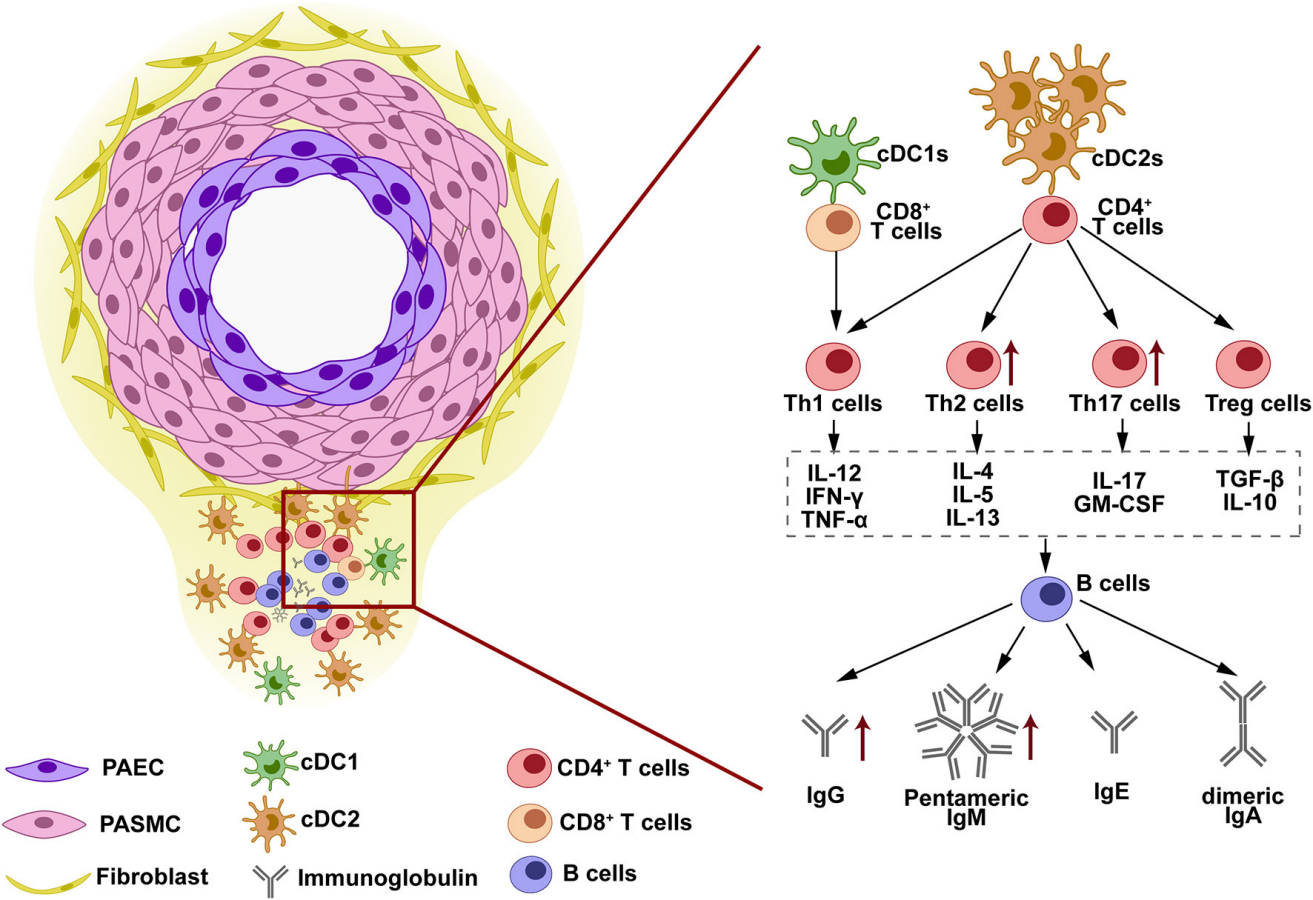
The structure and immune response in tertiary lymphoid organs (TLOs). TLOs are found around the pulmonary vascular lesion in the lung tissues of clinic pulmonary arterial hypertension (PAH) and experimental pulmonary hypertension (PH). The lymphoid structure of TLOs is constituted by dendritic cells (DCs) at the border, B cells and immunoglobulins at the core, and T cells at the interlayer. This structure facilitates the cell-cell interaction in TLOs. DCs are located in the outer layer of TLOs, closed to the remodeled vessels. Antigens exposed by vessels are phagocytized by DCs and presented to T cells. T cells are activated and differentiated into various subtypes when cross-presenting with DCs. The cytokines released by different T-cell subtypes determine the antibody class-switching destination of B cells when T-B cell interaction occurs. IgG, IgM, IgE, and IgA are released by B cells and can be detected in the lung tissues, serum, and BALF of PAH/PH subjects.

Tertiary lymphoid organs are structures observed in the lung tissues of clinic IPAH and experimental PH (49, 52). TLOs are similar to lymph nodes, locating around pulmonary vessels. The plasma cells constituted the core of TLOs structure with Th surrounded and DCs infiltrated in the border (49). The presence of TLOs suggested the immune cells as discussed above are not scattered in the lung tissue but have a close histological relationship instead. As described above, the formation of TLOs is the result of cells infiltration and retention. First, downregulation of CCR7 in PH subject block DCs homing. Then, activated DCs release cytokines that attract T cells and B cells, such as CCL19/21, lymphotoxin β receptor (LTBR), and CXCL13 (49, 52). Block DCs homing through CCR7 antagonism enlarged the size of TLOs in MCT-induced PH rats, while block B-cells infiltration through LTBR blockade decreased the size of TLOs (49). Experimental reduction of the TLOs also decreased the production of immunoglobulins in rat PH. This evidence suggests that the TLOs facilitate the cell-cell interaction during the adaptive response in the PH lung and are crucial for auto-antibody production and PH development (as shown in Figure 1).

## Auto-Antigen Exposure

Antigens mediate the pathogen recognition process and receive antibody binding in adaptive response. Therefore, identifying the local auto-antigens is crucial in IPAH and experimental PH. Vascular injury is considered the initiator of adaptive response in autoimmunity-involved vascular diseases (56, 57). Similarly, the pulmonary vascular lesion is considered as the repository of the auto-antigens in clinic IPAH or experimental PH. Experimentally stabilizing pulmonary vessels through Salubrinal decreased the TLOs formation and auto-antibodies production in MCT-induced rat PH (49). Among the cells constructed in the vessels, pulmonary endothelial cells and fibroblasts are the sources of auto-antigens in PAH. The identification of target antigens was revealed through the proteome approach, and the potential target antigen is listed in Table 1.

**Table 1:** Target auto-antigens identified in vessel cells from the patients with idiopathic PAH (IPAH).

**Target cell**	**Auto-antigens**	**Mainly functions**
Fibroblasts (58)	Vimentin, calumenin, phosphatidylinositol 3-kinase Tropomyosin 1 HSP 27, HSP 70, glucose-6-phosphate dehydrogenase	Cytoskeletal organization, cell contraction, oxidative stress.
Endothelial cells (59)	Lamin A/C, tubulin β-chain	Nuclear membrane, microtubules.
Smooth muscle cells (60)	Stress-induced phosphoprotein 1, α-enolase	Vascular contraction.

### Auto-Antigens in Fibroblasts

Approximately 40% of the patients with IPAH carrying anti-fibroblast antibodies (AFAs) suggest the pool of antigens exposed in fibroblast (58). Dr. Terrier identified 16 potential target antigens through MALDI-TOF MS in sera of human IPAH (58). These target antigens are mainly involved in the cytoskeletal organization (vimentin, calumenin, and phosphatidylinositol 3-kinase), cell contraction (Tropomyosin 1), and oxidative stress (heat shock protein (HSP) 27, HSP 70, and glucose-6-phosphate dehydrogenase) (58). Similarly, the auto-antigens expressed in the fibroblasts were observed in experimental PH (49). In both hypoxia-induced PH rats and MCT-induced PH rats, auto-antibody was specifically combined with pulmonary adventitial fibroblasts (49, 61), suggesting the auto-antigens exposure in fibroblasts.

### Auto-Antigens in Endothelial Cells

In patients with IPAH, serum IgG antibody showed higher intensity in binding with endothelial cells extracts, suggesting the potential target antigen exposed in pulmonary endothelial cells (62). The prevalence of anti-endothelial cell antibodies (AECAs) in the patients with IPAH was 62.1% in IgG isotype and 44.8% in IgM isotype (14), however, in small populations. Similarly, by proteomic approach, lamin A/C and tubulin β-chain were identified as the target of AECAs (59).

### Auto-Antigens in Smooth Muscle Cells

Dr. Bussone reported that the antigens exposed in vascular smooth muscle cells also attract auto-antibodies binding (60). They reported two targets identified through the proteomic approach, which are stress-induced phosphoprotein 1 and α-enolase (60). The detection rate of anti-smooth muscle cells antibody to stress-induced phosphoprotein 1 is 24% in the patients with IPAH (60).

## Auto-Antibodies Production

In PAH/PH lung tissues, B-cell infiltration and activation facilitate the local auto-antibodies production. Immunoglobulins contain Fab fragment and Fc fragment, which are combined with antigens and Fc receptors, respectively. The auto-antigens exposed by vascular cells have been discussed above. The Fc receptors vary with the Fc fragment embedded in different immunoglobulin isotypes. As the Fc receptors are expressed by different effector cells, the injury caused by immunoglobulins varies with the specific effector cells (Table 2).

**Table 2:** The function of auto-antibodies in PAH.

**Isotype**	**Present**	**Fab fragment targets**	**Roles**	**Fc receptors**	**Effector cells**
IgG	Blood of IPAH patients (58–60), experimental PH subjects (49, 61)	Fibroblasts, smooth smooth muscle cells and endothelial cells (58–60)	Induce pro-inflammatory cytokines release in fibroblasts and endothelial cells (49, 63, 64), Change phenotypes in fibroblasts (65), Induce apoptosis in endothelial cells (66), activate complement system (61)	FcγRI, FcγRII, FcγRIII	Monocytes, macrophages, dendritic cells, NK cells (67, 68)
IgM	Blood of IPAH patients (14), experimental PH subjects (61)	Endothelial cells (14)	Activate complement system (61), immune homeostasis (69, 70)	FcμR	B cells, T cells, NK cells (71)
IgE	BALF of experimental PH subjects (32)	Unclear	Attract effector cells	FcεRI	Mast cells (72)
IgA	Circulating plasmablasts of IPAH patients (51, 73)	Unclear	Attract effector cells	FcαRI	Neutrophils (74)

### IgG Isotype

IgG isotype is the preponderant population in immunoglobulins. Most auto-antibodies observed in clinic IPAH (such as AFAs and AECAs) are IgG isotypes (58, 62). In MCT-induced rat PH, auto-antibody in IgG isotype also showed elevation (49). Dr. Colvin collected the autoantibody-containing plasma from MCT-injected rat and transferred into native rats. Rats transferred auto-antibody showed severe PH (49), suggesting the detrimental role of auto-antibody in PH development. In the cell injury process, on one hand, IgG bind with the target antigens in fibroblasts or endothelial cells through Fab fragment (58, 62). These combinations cause phenotype changes in the fibroblasts or endothelial cells. In fibroblasts, AFAs positive IgG stimulation induces profibrotic and proinflammatory chemokines production (49, 63), such as CXCL1, CXCL8, IL-1β, and IL-6. Moreover, fibroblasts change into myofibroblasts phenotypes when received AFAs treatment (65), observed as increased α-SMA expression. In endothelial cells, the AECA positive IgG induces ECs apoptosis (66), highly express adhesion molecular and proinflammatory cytokines (64). The phenotype changes in fibroblasts and endothelial cells partially explained perivascular inflammation, endothelial cells dysfunction, vessel stiffness, and muscularization. On the other hand, the Fc fragment in IgG links the effector cells with the antigens. For IgG isotype, its Fc receptors contain FcγRI (CD64), FcγRII (CD32), and FcγRIII (CD16) (67, 68). These receptors are mainly expressed in macrophages, monocytes, NK cells, and DCs (67, 68). Macrophages and monocytes occupy a large population in the lung tissues, and they play a crucial role in PAH/PH development (5, 75). The infiltration of macrophages is observed in clinic PAH and experimental PH. The function of IgG stimulated macrophages is widely recognized as cytokines releasing [such as IL-6 and IL-10 (76)]. The DCs also expressed IgG receptor. DCs mediate T-cell activation upon IgG stimulation (77). IgG receptor FcγRII also expressed in endothelial cells and smooth muscle cells in the systemic circulation (68), whether it is expressed in pulmonary vascular cells is unknown. Although no direct evidence shows the similar function in IgG auto-antibodies extracted from PH subject, these research studies suggest that effector cells participate in the IgG auto-antibodies mediated vascular injury.

### IgM Isotype

Autoantibodies in IgM isotype were identified along with IgG isotype in the patients with IPAH (14). The present IgM isotype autoantibodies are mostly AECAs (14). The abnormal IgM is also seen in the experimental PH mice and rats, and it deposits in the luminal/medial area (61). These observations suggest that the IgM isotype mainly targets intima injury. IgM functions as activating complement system in pathophysiology. Due to its pentameric structure, it shows more powerful than the IgG isotype (78). The complement system is a newly identified participator in perivascular inflammation during PAH/PH development (79, 80). By proteomic analysis, the activation of complement cascades was discovered as one of the most upregulated signaling pathways in the early stage of PAH development (61). Similarly, the complement activation was also observed in the hypoxia-induced PH mice/rats, MCT-induced PH rats, and Sugen 5416 followed by hypoxia (SuHx)-induced PH rats. Moreover, the level of the complement component is correlated with clinical outcomes of patients with PAH (80). These observations suggest the essential role of the complement system in PAH/PH development. A previous study demonstrated that IgM and IgG are critical for the activation of the complement system in hypoxia-induced PH subjects (61). IgM deposition correlated with C4, suggesting its role in initiating classical and lectin pathways (61). Immunoglobulin deficient mice (μMT mice) failed to activate complement cascade, subsequently decreased the perivascular inflammation (61). Therefore, the IgM and IgG are the key initiator of complement cascade. The Fc receptor of IgM is named FcμR, and it is mostly expressed in lymphocytes (B cells, T cells, and NK cells in human; only B cells in mice) (71). The present knowledge of FcμR is based on *Fcmr-*ablated mice. *Fcmr-*ablated mice exhibited altered the subset of B cells, elevated IgM level, and dysregulated immune responses (69, 70), suggesting that IgM maintains immune homeostasis through FcμR. A polymeric immunoglobulin receptor (pIgR) is another receptor of IgM that binds with the J chain that is carried by polymeric immunoglobulins (such as pentameric IgM and dimeric IgA) (81). Th17 and IL-17 upregulated the pIgR expression (82, 83), however, the present studies show pIgR mostly expressed in the epithelial cells. Whether pIgR is expressed in leukocytes and whether pIgR participates in IgM mediated pulmonary vascular dysfunction needs further investigation.

### IgE Isotype

IgE elevation is commonly seen in allergic diseases or infectious diseases (84), however, in cardiovascular diseases, IgE also elevates. Serum IgE level increases in atherosclerosis, left heart failure, and abdominal aortic aneurysm (85–87). A variety of research studies indicate that IgE contributes to PAH/PH development. Ovalbumin (OVA) is an antigen commonly used in mice asthma models (31). OVA-stimulated mice exhibited pulmonary vascular remodeling without right ventricular systolic pressure (RVSP) elevation. The mice that received Sugen 5416 pre-injection showed severe PH when combined with OVA stimulation (88). Moreover, hypoxia combined with OVA-induced mice PH development (32). These observations indicate the contribution of IgE in PAH/PH development. IgE production occurs when B cells interact with Th2 cells (33, 35). As Th2 cells and Th2 response present in clinic PAH and experimental PH (31, 32), these facilitate B-cell class-switching into IgE isotype. Indeed, in the Th2 activation model, IgE elevated in bronchoalveolar lavage fluid (BALF) of PH subjects (32). Mast cells are one of the effector cell types of IgE, as it expresses the IgE high-affinity receptor FcεRI. FcεRI binds with IgE Fc fragment through its subunit (72). In clinic PAH and experimental PH, mast cells infiltrate and locate around pulmonary vessels (6, 89, 90). Mast cells inhibition prevents PH development in MCT-induced rats (11). Upon IgE stimulation, mast cells release vascular endothelial growth factors, IL-6, IL-13, and IL-33 (91). These growth factors and cytokines have been reported to contribute to perivascular inflammation and vascular remodeling (92–95). These results strongly suggest the association of IgE in PAH/PH development. Omalizumab is an IgE antagonist (96). Omalizumab is used in treating asthma through blocking IgE (96). Blocking IgE through Omalizumab attenuated left heart failure and abdominal aortic aneurysm (85). Therefore, Omalizumab therapy is a promising strategy in PAH/PH treatment once the contribution of IgE is demonstrated.

### IgA Isotype

Immunoglobulin A is the second most abundant isotype in serum immunoglobulins, but the most frequent subtype at the mucosal site (97). IgA that is enriched in the respiratory system and intestinal tract is named mucosal IgA, as it participates in host-pathogen defense in the mucosal lumen. Mucosal IgA is dimeric IgA (linked by J chain) while circulating serum IgA is monomeric (98). Serum IgA elevation is reported in severe PH associated with primary Sjögren's syndrome (73), and IgA-producing plasma cells are found in the blood of patients with IPAH (51). IgA Fc receptor FcαRI (CD89) is expressed by neutrophils. IgA activated neutrophils release leukotriene B4 (LTB4), which attract more neutrophils infiltration (74). Neutrophils infiltration and activation are observed in clinic PAH and experimental PH. The activated neutrophils released neutrophil extracellular traps (NETs), containing elastase, cytokines, chemokines, and proteases (7). These contents cause tissue damage and vascular disorder, such as ET1 release in PAECs and proliferation in PASMCs (7, 99). These results showed that IgA might induce neutrophil infiltration and activation in lung tissues of PAH/PH subject through Fc receptor FcαRI, subsequently induce tissue damage. Moreover, dimeric IgA also has a J chain structure (81). Therefore, whether pIgR participates in IgA mediated vascular dysfunction will be determined by the structure of IgA (dimeric or monomeric structure). Several strategies have been designed to block IgA and FcαRI to alleviate autoimmune injury. MIP8a, the anti-FcαRI mAb, reduced neutrophils activation, NETs formation, and tissue damage in rheumatoid arthritis (RA) patients (100, 101). Also, the peptides that mimic IgA or FcαRI sequences block the binding between IgA and FcαRI, decrease the infiltration of neutrophils induced by IgA (102).

## Discussion

Growing evidence indicates that adaptive response occurred in the lung tissues leads to local immunoglobulin production. These results support the observation that IPAH patients without autoimmune diseases also showed positive in auto-antibodies. The injury of auto-antibodies focuses on (a) vascular damage caused by antigen-antibody binding through Fab fragment; (b) leukocytes attraction and activation by Fc receptor. Moreover, the presence of TLOs and Th subtypes strongly suggested the B-cell activation and the diversity of immunoglobulin isotypes. IgE and IgA auto-antibodies might also exist besides IgG and IgM. Macrophages, mast cells, and neutrophils are known as participations in non-specific immunity. These innate immune cells are attracted by chemokines and activated by non-specific pathogens. Whether auto-antibodies are another attractors and activators for these effector cells warrant further investigation.

Based on clinical observation and experimental animal models, the production of auto-antibodies is both the cause and the consequence of IPAH development. Auto-antigens in pulmonary vessels should be phagocytized by APCs, which is the prerequisite in initiating the antibodies production. However, some experimental evidence is missing in this process: (a) if cell injury and cell death are needed in phagocytosis by APCs; (b) if antigen-presenting is stronger in mutation carriers. BMPR2 mutation promotes a pro-inflammatory and pro-apoptosis state in the endothelial cells (103). Whether endothelial cell apoptosis caused by BMPR2 mutation triggered APCs phagocytosis needs further investigation. The present studies demonstrated that AECAs promote adhesion and inflammation (64), other than the proliferation (66) in normal endothelial cells. Whether AECAs cause more severe vascular dysfunction in mutation carriers are unknown. Considering the incomplete penetrance in BMPR2 mutation, we hypothesize auto-immunity acts as an additional trigger that participates in disease progression in unaffected mutation carriers.

Remarkably, the auto-antibodies identified in patients with IPAH showed some similarities and differences with those in PAH with connective tissue diseases (PAH-CTD). Antigen targets are the most obvious differences. For the patients with IPAH, the antigen targets are exposed in pulmonary vessels, such as Lamin A/C in endothelial cells (Table 2), however, the targets in PAH-CTD are commonly nuclear (identified as antinuclear antibodies) or DNA (identified as anti-double-stranded DNA antibodies) (104). Then, the inner relationship between auto-antibodies and PAH is different. In PAH-CTD, auto-antibodies heavily deposit in the connective tissues prior to the lung. PH is secondary to connective tissue diseases. However, for the patients with IPAH, auto-antibodies impaired pulmonary vessels first, while other tissues impairment is less reported. What is in common is that, autoantibodies reported in two kinds of PAH aggravate pulmonary vascular dysfunction. AECAs purified from IPAH and PAH-CTD show pro-inflammatory and pro-adhesive effects in endothelial cells, however, only AECAs from PAH-CTD promote proliferation in endothelial cells (64, 66). AFAs purified from the patients with SSC induced fibroblast activation (58). Auto-antibodies targeting the smooth muscle cells induced the contraction of cells (60). Auto-antibodies found in the experimental PH models activated complementary systems and aggravated inflammation in the PH lung (61).

Anti-inflammatory therapy has been evaluated for years. IL-6 is one of the most promising targets in treating the PAH (9). A pre-clinical study showed blocking IL-6 is effective in attenuating PH development in MCT rats, SuHx rats, and hypoxic mice (9, 95, 105); however, the clinical trials of Tocilizumab (an IL-6 receptor antagonist) is less effective than expected (106). Other targets are also considered, such as TNFα (etanercept) and IL-1 (anakinra). Of note, the anti-inflammatory drugs are feeble in suppressing the production of the immunoglobulin (4, 14), therefore, the other strategy should be taken to decrease the vascular injury caused by auto-antibodies. For IgE and IgA, which are less important in maintaining host-defense, targeting the immunoglobulins themselves (such as Omalizumab and targeting IgE) or their Fc receptors (such as anti-FcαRI mAb and targeting Fc receptor of IgA) might be effective in blocking autoimmune response. More directly, targeting B cells might limit the activation of B cells and slash the production of aberrant immunoglobulins. Rituximab is an anti-human CD20 type I chimeric antibody. Blocking CD20 by rituximab inhibits the proliferation of stimulated B cells. Rituximab is used to treat certain autoimmune diseases through decreasing auto-antibody production. The clinical trial revealed rituximab improved 6MWD in patients with PAH-CTD (NCT01086540). Whether restricting B cells affects IPAH development and whether rituximab is listed as first-line treatment will be uncovered in the near future.

## Author Contributions

TS wrote the manuscript. YX revised the manuscript. JW conceived the framework. All authors contributed to the article and approved the submitted version.

## Funding

This study was financially supported by the National Key R&D Program of China (Grant no: 2019YFA0801703) and the National Natural Science Foundation of China (Grant no: 82090011).

## Conflict of Interest

The authors declare that the research was conducted in the absence of any commercial or financial relationships that could be construed as a potential conflict of interest.

## Publisher's Note

All claims expressed in this article are solely those of the authors and do not necessarily represent those of their affiliated organizations, or those of the publisher, the editors and the reviewers. Any product that may be evaluated in this article, or claim that may be made by its manufacturer, is not guaranteed or endorsed by the publisher.
